# An objective and cross-sectional examination of sun-safe behaviours in New South Wales primary schools

**DOI:** 10.1186/s12889-016-3917-9

**Published:** 2017-01-05

**Authors:** Dean A. Dudley, Wayne G. Cotton, Matthew J. Winslade, Bradley J. Wright, Kirsten S. Jackson, Alexandra M. Brown, Vanessa Rock

**Affiliations:** 1 Department of Eduational Studies, Faculty of Human Sciences, Macquarie University, North Ryde, NSW 2109 Australia; 2 Faculty of Education and Social Work, University of Sydney, Sydney, NSW 2001 Australia; 3 School of Teacher Education, Faculty of Education, Charles Sturt University, Bathurst, NSW 2795 Australia; 4 Skin Cancer Prevention Unit, Cancer Council New South Wales, Woolloomooloo, NSW 2011 Australia

## Abstract

**Background:**

Previous evaluations have supported the link between sun protection policies and improved sun protection behaviours. However these evaluations have relied on self-reported data.

**Methods:**

A cross-sectional design as part of an ongoing 18-month cluster-controlled trial in primary schools (*n* = 20) was used. Researchers conducted direct observations to record students’ hat use and teachers’ use of sun protective measures during recess and lunch. Researchers also recorded the volume of sunscreen consumed in each school.

**Results:**

Only 60% of primary school children wear a sun-safe hat during their breaks when observed using objective measures. Weak correlations were observed between the wearing of a sun-safe hat and a school’s socio-economic status (*r* = 0.26). All other independent variables measured had only very weak correlations (*r* < 0.19) with sun-safe hat wearing behaviour of students. Sunscreen consumption by school students during the school day is negligible.

**Conclusions:**

A large percentage of NSW primary schools in this study wear sun-safe hats during the school day but this is well below what has been reported in previous national surveys. Given the finite resources of schools and the correlation, though small, with SES status for these behaviours, it behoves researchers to investigate low-cost solutions to these problems. Further qualitative data will also be needed to inform the enablers and barriers for sun-safe behaviour interventions to be adopted in NSW primary schools.

## Background

Currently, two out of three Australians will develop some form of skin cancer before the age of 70 [1]. In 2010 there were over 778,000 for non-melanoma skin cancer and 11,545 new cases of melanoma diagnosed. More than 2000 people die from skin cancer every year [2] and 99% of non-melanoma skin cancers and 95% of melanomas are caused by overexposure to solar ultraviolet (UV) radiation [3].

Protecting skin from exposure to UV radiation is the simplest and most effective way to prevent skin cancer [3, 4]. Research suggests that reducing children’s exposure to UV radiation, particularly in the first 15 years of life, significantly reduces their risk of developing skin cancer later in life [4] The World Health Organization’s (WHO) *Sun Protection and Schools* report [5] outlines that schools need to implement both structural and organisational strategies to ensure sustainable sun protection for students and staff.

In New South Wales (NSW) approximately 650,000 children aged between five and 12 years spend up to 7 h per day, up to 40 weeks per year attending primary school [6]. As students participate in both organised and recreational outdoor activities during peak UV times, the NSW Department of Education provide schools with sun protection guidelines [7] and recommend they join Cancer Council NSW (CCNSW) SunSmart Program [8].

The SunSmart Program, launched in NSW in 2008, is based on the Health Promoting Schools principles [9] and supports childcare services and schools to develop and implement a comprehensive sun protection policy [8]. To be eligible for SunSmart membership, schools must submit a sun protection policy addressing 10 recommendations within three key areas: influencing the environment, modifying behaviour and integrating education [8]. To maintain SunSmart membership, schools must review and resubmit their sun protection policy to CCNSW every 3 years [8]. The SunSmart Program has been evaluated in three phases over the last 10 years via national survey of Australian primary schools’ sun protection policy and practices that monitors trends in primary schools’ sun protection policies and practices, and provides comparisons between SunSmart and non-SunSmart schools across Australia [10, 11]. Results demonstrated improved sun protection practices being adopted in SunSmart primary schools compared to non-SunSmart schools. This included them having more comprehensive sun protection policies and practices, sport lessons being held earlier in the day to avoid peak UV times and parents being provided with sun protection information [11].

As of April 2015, 78% (*n* = 1983) NSW primary schools were members of the SunSmart Program. The national survey identified opportunities for strengthening sun-safe behaviours of primary school children attending SunSmart schools. This included increasing the number of students wearing sun-safe hats, the availability and promotion of sunscreen and an increase in staff role-modelling sun-safe behaviours themselves [5].

These three behaviours were identified for inquiry in this study as evidence has shown the wearing of sun-safe hats, clothing and sunscreen in children has the potential to reduce the risk of skin cancer [4]. There is some research to suggest that adult role modelling may also influence a child’s sun protection behaviours [12, 13]. In any case, an effective intervention to modify these behaviours would need to translate to school health policy that enables practical application, with little cost to the school.

Past evaluations of primary school sun protection policies have found that there is a lack of data supporting the link between sun protection policies and observations of sun-safe behaviour [14]. A recent observational evaluation in primary schools has been conducted in the state of Queensland to address this [13]. With approximately half of all students and adults observed wearing hats, it was identified that there is room for improvement in hat wearing and role modelling behaviour of students and adults.

The SunSmart Evaluation and Policy Intervention Study aims to collect and measure objective data around student use of sun-safe hats, student sunscreen consumption, and adult role modelling of sun-safe behaviours in primary schools. This study will build on the above findings through observing students and teachers located in primary schools in the state of NSW, and identifying opportunities to develop and implement a policy-based intervention.

This study reports on objective baseline data collected as part of a randomised cluster controlled intervention to assess students’ use of sun-safe hats and sunscreen, and teacher role modelling of sun-safe behaviours in NSW primary schools.

## Methods

### Study design

The SunSmart Evaluation and Policy Intervention Study is an 18-month primary school-based intervention and is being evaluated using a cluster randomised controlled trial. The rationale and study protocol have been published previously [15]. This paper reports on the baseline data of that study and their analyses. Ethics approval was obtained from an Australian University Human Ethics Committee (HREC: 2014/062) and the New South Wales Department of Education (SERAP: 2014148). The SunSmart Evaluation and Policy Intervention Study is registered with the Australian and New Zealand Clinical Trials Registry (ACTRN12614000926639).

Following the initial recruitment processes, researchers conducted baseline assessments at participating schools. Principals provided written informed consent in order for their schools to participate in the study and for the results to be published in peer-reviewed journals.

### Recruitment and study participants

Schools recruited to the study were government primary schools and a current member of the SunSmart Program in the Greater Western Sydney Region, NSW, Australia (approx. 33.75 deg S,150.70 deg E). All eligible schools (*n* = 167) were sent an initial email with an invitation to participate in the study. CCNSW and the researchers identified a short-list of schools that may be receptive to participating in the study based on their response to the recruitment email (*n* = 40). Schools that were short-listed schools were pooled and received a follow up call from the project researchers to ascertain whether they would like to participate in the study. A power calculation was conducted to determine the sample size and number of observations required, which resulted in the first 20 schools that demonstrated interest being recruited into the study.

Trained research assistants (RAs) conducted all assessments. All RAs completed training sessions prior to assessment to maintain consistency and the same RAs were used during the data collection phase.

### Outcomes and instruments

The primary outcome variable of this study was the wearing of sun-safe hats during break periods of the school day (i.e. recess and lunch) by students. Whilst sun-safe behaviours have been observed in a beach setting [16, 17] these were deemed inappropriate for school settings due to the variables being observed (i.e. type of swimwear). There have been a few studies to date that used a mix of surveys and on-site evaluations of SunSmart programs in Hawaii (Glanz et al, [18]) and observations of public recreation venues (Dobbinson et al, [19], Nikles & Harrision, [20]). Very few however record sun-safe behaviours of children in school settings.. In this study, we adapted the System for Observing Play and Recreation in Communities (SOPARC) [21] to capture sun-safe behaviour data in children. The SOPARC [21] is based on momentary time sampling techniques in which systematic and periodic scans of individuals and contextual factors within predetermined target areas are made. Computer tablets (Apple Inc, USA) installed with the iSOPARC Application Version 1.75 (CIAFEL, Portugal: https://ciafel.fade.up.pt/isoparc/) were used to provide an objective measure of hat wearing by children during recess and lunch.

In the traditional application of the iSOPARC tool, a scan of each subject is electronically coded and identified by: sex (male or female), intensity of activity (Sedentary, Walking, or Very Active), and whether they are a Child, Teen, Adult or Senior. For this study, given all the subjects were children, the third battery of coding (Child, Teen, Adult or Senior) was changed to detect whether the student was Unprotected (i.e. wore no hat), Partially-Protected (i.e. was wearing a baseball cap), Fully-Protected (i.e. was wearing a sun-safe hat: broad-brimmed, bucket hat or legionnaire).

Separate scans were made for females and males, and entries are also made for time of day, temperature, UV radiation level, area accessibility, area usability and presence of supervision. Each observation was conducted twice during the recess and lunch breaks for both females and males. A single scan involved a researcher observing each student, of one sex, within a pre-defined Target Area sequentially from left to right without pausing.

Direct observations were made in the designated Target Areas that had been identified by school principals (or their proxy) as areas that were likely to provide opportunities for students to have sun exposure during their recess and lunch periods (i.e one shaded and one unshaded play area). It is important to note here that shaded areas had to be man-made structures and were all metal structures with solid roofing. No areas were covered with ‘shade cloth’ or other permeable materials.

Additional data recorded prior to the direct observation scans included; temperature and UV level at the start and end of the observation period; whether the observation was made at recess or lunch; start and finish times of recess and lunch; and whether the area was shaded or not.

Researchers also recorded whether the teacher supervising the children’s behaviour in the Target Area was role-modelling sun-safe behaviour. An observation note was added to the final iSOPARC data on whether the teacher was wearing a) a sun-safe hat; b) sunglasses or other appropriate eye protection (i.e. transition lensed optical glasses; and c) a sleeved shirt and collar. As there was no more than 4 teachers present during any observation period, research assistants were able to record all of the teacher’s sun protective behaviours. Student clothing was not recorded as State requirements are that all students wear a school uniform that requires a collared shirt that covers the shoulders and upper arm as a minimum.

Thirty two field-based inter-rater reliability checks were conducted during the 10-week observation period. During reliability checks, two observers independently coded the same students in the same lunch or recess period. A high degree of reliability was found between measurements. The average measure Intraclass Corelation Coefficient (ICC) was .912 with a 95% confidence interval from .885 to .932 (*F* = 11.324, *p* < .001).

In an effort to minimise bias, the inter-rater reliability checks on 4% of the iSOPARC observations were randomised in order to prevent possible collusion. Recess and lunch break observations were randomly selected and observers and schools were given limited notice of when a reliability check was going to occur (usually less than 24 h).

Sunscreen consumption was also recorded during the observation period. All Stage 3 (i.e. Grades 5–6 or children aged 10–12 years) classes within the 20 schools were given accurately weighed and filled one-litre sunscreen containers (1 l = 1.07kg) at the start of the period. These containers were placed near the door of the students’ classroom. The containers were removed at the end of the observation period and weighed again. This consumed weight of sunscreen was divided by the number of students within the class to produce a baseline consumption rate per student.

Demographic data including socioeconomic status based on postcode of the school (Socio-Economic Indexes for Areas—SEIFA) [22] and national school enrolment data collected by the federal government (Index of Community Socio Educational Advantage—ICSEA) [23] was recorded for each school.

The quantitative variables reported in this paper include: student sex, student hat wearing behaviour, teacher sun safety behaviour, environmental factors, school SEIFA and ICSEA status, and student activity levels. Student sex was reported as male or female. Student hat wearing behaviour was reported as a) no hat; b) baseball cap; c) legionnaire hat; d) 360° broad brimmed or bucket hat; or e) sun-safe hat which was the sum of (c) and (d). Teacher sun safety behaviours were reported as a) hat wearing; b) sunglass wearing; c) sun protective clothing (i.e. covered shoulders and collar); and d) shade seeking.

The remaining quantitative variables were reported as correlation alphas to student wearing, or not wearing, a sun-safe hat during recess or lunch break. Environmental factors were shaded area (dichotomous), UV levels and temperature (continuous). The school factor was the school ICSEA value (continuous) and student enrolment numbers. Student activity levels were the proportion of time spent in each of the activity levels (Sedentary, Walking, or Vigorous).

Percentages were for the entire sample and then stratified by sex. Pearson and bi-serial correlations were then calculated for the continuous and dichotomous variables accordingly. This was done to ascertain the relationship between the primary outcome variables and the covariates at the smallest discernible level. All data were analysed using Statistical Package for Social Science (SPSS) version 22.

## Results

The first round of data collection occurred as planned in school Term 4, 2014 (September-December). The collection involved teams of two RAs randomly visiting the 20 consenting schools three times. The RAs each observed a separate predetermined covered and sun exposed playing area during recess and again during lunch time at each school. This resulted in 240 potentially observable sessions, of which 238 were completed. One session (i.e. two observations) were cancelled due to wet weather on the last observation day of the term. During each observable session, data was collected twice for male students in the target areas and twice for female students. It should be noted that when there were no males or no females in the playing area, observations were not recorded for that sex. This resulted in 839 individual observations being recorded out of a possible 960 (87%).

### Demographic Data

An overview of the demographic characteristics of the schools involved in the study can be seen in Table 1.

**Table 1:** Demographic characteristics of the 20 schools involved in the study

		Baseline
Schools stratified by SEIFA Index (% of schools) based on postcode of school		
	1	0 (0%)
	2	1 (5%)
	3	3 (15%)
	4	5 (25%)
	5	11 (55%)
Distribution of school students stratified by the ICSEA		
	Bottom Quarter	30.6%
	Middle Two Quarters	49.8%
	Top Quarter	19.6%
Total student enrolment in schools		*n* = 7971
By Sex		
	Boys	*n* = 4082
	Girls	*n* = 3886

Table 1 provides an overview of the 20 schools that consented to being in the study. The tables shows that 55% of the schools participating in the study (*n* = 11) are located in the 5th decile (highest) of the Socio-Economic Indexes for Areas (SEIFA), with the remaining schools being in the lower deciles. This trend is highlighted when the students in the schools are stratified by the Index of Community Socio-Educational Advantage (ICSEA). This analysis reveals that 30.6% of the students enrolled in the participating schools are in the bottom quarter of the Australian population, with only 19.6% being in the top quarter.

### Main Results

Table 2 reports the unadjusted means and standard deviations of the students and teachers observed during the 240 observable sessions occurring during recess and lunch breaks in Term 4, 2014 (September—December).

**Table 2:** Unadjusted means and standard deviations for student and teacher factors of sun safe behaviour

Category	Recess & lunch behaviour (%) (*n* = 839)		Recess & lunch behaviour (%) Males Obs. (*n* = 420)		Recess & lunch behaviour (%) Females Obs. (*n* = 419)	
	M (n)	SD	M (n)	SD	M (n)	SD
Student Hat Wearing Behaviour						
No Hat	19.3 (162)	26.0	15.0 (63)	21.2	23.8 (99)	29.5
Baseball Cap (No side brim or back flap)	20.5 (172)	33.7	24.5 (103)	35.8	16.5 (69)	31.1
Legionnaire hat	10.6 (89)	26.2	12.1 (51)	27.2	9.1 (38)	25.0
360° Brimmed hat (Bucket or Broad)	49.6 (416)	41.8	48.3 (203)	42.5	50.9 (213)	41.1
Sun-safe hat^	60.1 (504)	40.1	60.2 (253)	41.2	59.9 (251)	39.1
Teacher sun-safe behaviour (%)	M (n)					
Number of observations (*n* = 514) with:						
Hat wearing behaviour						
- Sun-safe hat^a^	35.2 (181)					
- Baseball cap	11.1 (57)					
- No hat	53.7 (276)					
Sunglass wearing	69.9 (359)					
Sun protective clothing	50.8 (261)					
Shade seeking	49.0 (252)					

^a^Sun-safe hat is the sum of the peak cap with back flap and 360° brimmed hat

The table shows that 839 observations were conducted during these recess and lunch breaks with 60.1% (*n* = 504) of the students seen wearing a sun-safe hat (i.e. either a legionnaire hat or a 360° broad-brimmed or bucket hat). The table also shows that 19.2% (*n* = 161) of the students were wearing no hat at all during these times.

Table 2 also highlights that there is a substantial difference between student hat wearing behaviours of male and female students, whilst 19% students did not wear any form of hat during recess and lunch, 24% (*n* = 99) of female students would not wear a hat, compared with 15% (*n* = 62) of male students. However, the differences of wearing of sun-safe hats between the sexes during recess and lunch was consistent at 60% across both groups.

Further analysis of these results not revealed in this table but in the data showed that in 55% (*n* = 11) of the schools more than 80% of the students were wearing a sun safe hat, however the results also showed that in 20% (*n* = 4) of the schools less than 7% of the observed students were wearing a sun safe hat. This goes some way to our understanding of the amount a variance being reported in this table.

The lower section of Table 2 displays the sun-safe behaviours of the teachers on playground duty during the observational periods. Only teacher behaviour was observed because in NSW, primary school students are required to wear a school uniform. As such, very little, if any, variability in their clothing is evident. Teacher on the other hand are able to exercise choice in this outcomes with 35% of the teachers observed wearing a sun-safe hat (but 54% wearing no hat at all) and 70% of the teachers were observed wearing sunglasses. Sun protective clothing was defined if the teacher wore clothing with a collar and covered their shoulders and upper arm to the mid-bicep. Only 51% of teachers wore items of clothing that met this description when observed during recess and lunch playground duties.

In terms of sunscreen consumption, of the 141 one-litre containers of sunscreen issued to schools for use during this study, only 57 had their tamper seals broken. Of the 57 opened containers, only 5 containers exceeded more than 20% on the contents being consumed. The mean consumption per opened container per school ranged between 0 and 100g for 18 of the 20 schools. One school consumed an average of 130g per opened container (*n* = 2) and another school consumed 280g per opened container (*n* = 1).

A series of Pearson product-moment correlation coefficients were computed to assess the relationship between the wearing of sun safe hats and a range environmental, school, teacher and student behaviours. We have described the strength of the correlation using the guide that Evans [24] suggests for the absolute value of *r* being: .00-.19 “very weak”; .20-.39 “weak”; .40-.59 “moderate”; .60-.79 “strong” and; .80-1.0 “very strong”. A summary of these correlations can be seen in Table 3 where they are also displayed by sex.

**Table 3:** Correlation of varying recess and lunch break contexts with the wearing of sun safe hats by sex

	Males and Females Observations (*n* = 839)		Male Observations (*n* = 420)		Female Observations (*n* = 419)	
	r	*p* value	r	*p* value	r	*p* value
Environmental factors						
Temperature	0.00	0.91	0.02	0.71	0.03	0.58
UV Index	0.04	0.20	0.09	0.08	0.00	0.99
School factors						
School ICSEA	0.26	<0.01^**^	0.28	<0.01^**^	0.23	<0.01^**^
Teacher on duty factor						
Teacher wearing hat	0.15	<0.01^**^	0.14	<0.01^**^	0.16	<0.01^**^
Student activity levels						
Sedentary	0.03	0.42	0.00	0.99	0.06	0.23
Walking	0.12	<0.01^**^	0.14	<0.01^**^	0.10	0.04^*^
Vigorous	0.09	<0.01^**^	0.13	<0.01^**^	0.04	0.42

*r* Pearson correlation, *ICSEA* Index of Community Socio-Educational Advantage

^*^ Statistically significant at <0.05

^**^ Statistically significant at <0.01

There were only “very weak” and no statistically significant correlations found between the wearing of sun-safe hats and daily temperature or UV Index. However, there was a “weak” positive correlation (*r* = 0.26) between a school’s ICSEA and students wearing of sun-safe hats. Whilst this was statistically significant at *p* < 0.01, the two variables only had a shared variance of 6.8%. The results did not significantly vary when analysed further by the sex of the students observed when ICSEA was the independent variable.

The analysis found only “very weak”, though statistically significant correlations, between the teacher wearing of a sun-safe hat and the students wearing of sun-safe hats. The analysis also revealed that in only 10% (*n* = 2) of the schools, teachers modelled hat-wearing behaviour more that 65% of the time. Again, only “very weak” though statistical significant correlations were observed between sun-safe hat wearing by students and when they engaged in vigorous or walking levels of physical activity.

Consumption of sunscreen by students in Stage 3 was negligible. Cancer Council Australia recommends children apply 25ml per limb, on the head and neck, and on the torso (reference), which is equates of 25g of sunscreen. An analysis of students’ consumption of sunscreen during the 9-week observation period found that each student applied an average of 9.9 ml (SD = 18.61) of sunscreen. No significant or meaningful correlation was found to exist between a school’s ICSEA, daily temperature and UV Index or student activity levels with their students’ consumption of sunscreen.

## Discussion

The purpose of this study was to assess students’ use of sun-safe hats and sunscreen, and teacher role modelling of sun-safe behaviours in NSW primary schools. The main findings of the study were that 60% of students wear sun-safe hats (i.e. a legionnaire hat, a broad brimmed or a bucket hat) during recess and lunch periods, while 20% wear a baseball cap and 19% of students do not wear any type of hat during these times. Current national survey data on self-reported sun-safe hat wearing by primary school children as reported by school Principals states that 80% of students wear sun-safe hats during recess and lunch periods [11]. This 20% discrepancy appears indicative of the contrasts between self-report and objective guideline adherence to public health recommendations for school-aged children [25]. We recognise that self-reported instruments still remain a practicality in large-scale studies, however, we hope this study provides some further evidence for the need to validate self-report data from large-scale studies with at least some additional objective measures to understand the degree of bias that is possibly being reported in such studies.

When examining the data further, a weak positive relationship was found between a school’s ICSEA and the sun-safe hat wearing behaviours of its students. No relationship was found between the teachers and students sun-safe behaviours. This is of interest considering it has been established that children’s behaviours are influenced by what they observe and learn [26]. CCNSW’s SunSmart policy [27] requires that schools ask their staff to role model good sun-safety behaviours, including the wearing of sun-safe hats when they are outside. Similar findings were reported in the observational research conducted by Turner and colleagues [14] which noted schools may assume teachers are acting as role models without being asked, resulting in schools potentially placing less importance on this component.

To date, CCNSW has no data pertaining to the amount of sunscreen students consumed during school hours. Consumption of sunscreen by Stage 3 students in this study was negligible suggesting that these recommendations were not met. External incentives have been recommended as a motivator to improve compliance with SunSmart policy recommendations and sun-safe practices in schools [13]. As this study has identified opportunities to improve sun-safe us, the use of incentives will be considered moving forward for the intervention phase of this trial.

### Strengths and limitations

Strengths of this study are the high rate of observations recorded (87%). This was partially attributed to favourable weather conditions that meant only two observations were missed due to inclement weather. This appears to be one, of only a few objective studies of sun-safe behaviours and even fewer of those conducted within school premises and during the school day. We imagine that this should allow for a clearer understanding of routine sun protection practices that may be occurring. Earlier observation studies conducted by Turner and colleagues [13, 14] occurred from outside the school premises.

The main limitation of this study was that it reports findings from a number of schools within a relatively small geographic area. Future studies need to recruit more schools and use diverse geographical settings that are more indicative of Australian population demographics. Other limitations are that it reports on cross-sectional data. Further longitudinal research is needed to ascertain whether trends in these reported behaviours are consistent. Another limitation is that data were only collected over a 3-month period of the academic year. As such, we were unable to determine with these data if annual sessional issues, such as weather, influence these findings to a greater extent.

Furthermore, children were not directly observed using the sunscreen dispensers. So who, when or how the sunscreen dispensers were utilised remains a possible limitation.

## Conclusions

Although it appears that a large percentage of NSW primary school students wear sun-safe hats during recess and lunch breaks, it is apparent that this varies considerably across schools and it is well-below the percentages being reported in national surveys as a whole [10, 11]. It is also apparent that sunscreen use among certain students may be well below current health recommendations [27]. This study identifies specific areas within school sun-protection practices that could benefit from an intervention that promoted both the wearing of sun-safe hats and sunscreen. Given the finite resources of schools and the correlation, though small, with ICSEA for these behaviours, it behoves researchers to investigate low-cost solutions to these problems. Further qualitative data will also be needed to inform the enablers and barriers for sun-safe behaviour interventions to be adopted in NSW primary schools.

## Abbreviations

ACTRN: Australian and New Zealand Clinical Trials Registry Number
CCNSW: Cancer Council NSW
CONSORT: Consolidation Standards of Reporting Trials
HPS: Health Promoting Schools
NSW: New South Wales
NSWDEC: New South Wales Department of Education and Communities
SCT: Social Cognitive Theory
SERAP: State Education Research Approvals Process
SOPARC: System for Observing Play and Recreation in Communities
SPSS: Statistical Package for the Social Sciences
UV: Ultraviolet
WHO: World Health Organization
